# Circular RNA circNFKB1 promotes osteoarthritis progression through interacting with ENO1 and sustaining NF-κB signaling

**DOI:** 10.1038/s41419-022-05148-2

**Published:** 2022-08-09

**Authors:** Su’an Tang, Xiaoyu Nie, Jianzhao Ruan, Yumei Cao, Jingliang Kang, Changhai Ding

**Affiliations:** 1 Clinical Research Centre, Zhujiang Hospital, Southern Medical University, 510280 Guangzhou, Guangdong China; 2 Centre of Orthopedics, Zhujiang Hospital, Southern Medical University, 510280 Guangzhou, Guangdong China; 3 Department of Rheumatology and Immunology, Zhujiang Hospital, Southern Medical University, 510280 Guangzhou, Guangdong China; 4 Menzies Institute for Medical Research, University of Tasmania, 7000 Hobart, Tasmania Australia

**Keywords:** Osteoarthritis, Cell signalling, RNA

## Abstract

Inflammatory cytokines-induced activation of the nuclear factor κB (NF-κB) pathway plays a critical role in the pathogenesis of osteoarthritis (OA). Circular RNA (circRNA) has been identified as important epigenetic factor in numerous diseases. However, the biological roles of inflammation-related circRNAs in regulating OA pathogenesis remain elusive. Here, we revealed circRNA expression profiles in human primary chondrocytes with interleukin-1β (IL-1β) stimulation by circRNA sequencing. We identified a highly upregulated circRNA, termed as circNFKB1 in inflamed chondrocytes and osteoarthritic cartilage. As a circRNA derived from exon 2–5 of NFKB1, circNFKB1 is located in both cytoplasm and nucleus of chondrocytes. Furthermore, knockdown of circNFKB1 inhibited extracellular matrix (ECM) catabolism and rescued IL-1β impaired ECM anabolism whereas ectopic expression of circNFKB1 significantly promoted chondrocytes degradation in vitro. Moreover, intraarticular injection of adenovirus-circNFKB1 in mouse joints triggered spontaneous cartilage loss and OA development. Mechanistically, circNFKB1 interacted with α-enolase (ENO1), regulated the expression of its parental gene *NFKB1* and sustained the activation of NF-κB signaling pathway in chondrocytes. Therefore, this study highlights a novel ENO1-interacting circNFKB1 in OA pathogenesis, and provides valuable insights into understanding the regulatory mechanism of NF-κB signaling in chondrocytes and a promising therapeutic target for the treatment of OA.

## Introduction

Osteoarthritis (OA) is the most prevalent degenerative joint disorder with clinical symptoms characterized by joint pain, deformity, and dysfunction [1]. The pathological changes of OA involve the whole joint structures, mainly manifested as cartilage destruction. With the increasing aging and obesity of the population worldwide, the incidence of OA increases rapidly, bringing heavy medical burden to the society and individuals [2]. Unfortunately, due to the lack of disease-modifying therapeutics, the early treatment of OA is not satisfactory at present [3]. Therefore, further studying OA pathogenesis and exploring effective treatment strategies is of great value.

Overactivity of nuclear factor kappa B (NF-κB) signaling pathway has been linked to the pathogenesis of OA [4, 5]. Canonical NF-κB signaling is known to promote the expression of matrix metalloproteinases (MMPs) and suppress the expression of anabolic genes including Aggrecan (ACAN) and Collagen type II Alpha (COL2A1), ultimately causing the imbalance of chondrocyte extracellular matrix (ECM) metabolism. Blocking NF-κB activity via novel NF-κB inhibitor or intraarticular delivery of NF-κB siRNA significantly attenuates chondrocyte degradation and suppresses synovial hyperplasia in vivo [6, 7]. Hence, investigating the regulation of NF-κB signaling in OA progression would provide potential therapeutic target for OA therapy.

Circular RNAs (circRNAs) are covalently closed RNA transcripts generated by back-splicing of precursor mRNAs, which are more stable than linear RNAs without 5′ caps or 3′ poly(A) tail [8]. Due to a multitude of RNA-splicing patterns, circRNA could be derived from exons, introns, or exon-introns in eukaryotic cells [9]. Growing evidences demonstrate that circRNAs exhibit essential functions in various biological processes, including transcription regulation, protein post-translational modification, and signaling activation [10]. In addition, circRNAs are also widely involved in multiple pathological conditions, such as metabolic disturbance, tumorigenesis, and immune disorders [11–13]. Of note, aberrant expression of circRNAs contributes to musculoskeletal degenerative diseases [14]. Several OA-related circRNAs have been reported recently, indicating that these newly discovered RNAs as important epigenetic factors play crucial roles in OA pathogenesis [15–17]. However, these studies mainly focused on the biological functions of circRNA in OA progression, whether there are circRNAs regulating NF-κB signaling in OA remains largely unclear.

α-Enolase (ENO1) is an enolase isoform extensively expressed in almost all mammal cells, originally characterized as a key glycolytic enzyme [18]. In addition to serving as enzyme in energy metabolism, ENO1 has also been unexpectedly identified as an RNA-binding protein with moonlighting functions [19]. Intriguingly, as a member of the RNA degradosome in *Escherichia coli*, ENO1 is recently reported to mediate RNA decay not only in prokaryotes but also in eukaryotic cells [20, 21]. Compelling studies have recently revealed that ENO1 associates with noncoding RNAs in regulation of cancer development [22–24]. In inflammatory arthritis, enhanced surface expression of ENO1 in patient-derived peripheral blood mononuclear cells facilitates inflammatory response [25]. Furthermore, ENO1 in synovial fibroblast regulates cell proliferation and survival under hypoxia [26]. Nevertheless, whether there is an ENO1-interacting circRNA and its relevance to chondrocyte ECM metabolism remains elusive.

In this study, we endeavored to investigate the role of inflammatory cytokines-induced circRNAs in OA pathogenesis and their underlying mechanisms. We identified a functional ENO1-interacting circRNA (has_circ_0008012), termed circNFKB1, that promoted chondrocytes ECM degradation in vitro and in vivo. Mechanistically, circNFKB1 interacted with ENO1 and enhanced NFKB1 expression, ultimately activating the NF-κB signaling. We believe that our findings deepen our understanding of the complex regulation of NF-κB signaling and provide a new therapeutic target for OA.

## Materials and methods

### Human cartilage

This study has been approved by the Ethics Committee of Zhujiang Hospital, Southern Medical University. Human knee OA cartilage tissues were collected from patients with a history of knee OA after total knee replacement surgery (*n* = 30) undergoing arthroplasty in Zhujiang Hospital of Southern Medical University. All participants provided written informed consent before the operative procedure. Rheumatoid arthritis and other joint diseases that did not meet the study criteria, such as malignancies, were excluded. Basic information including age, gender, weight, height, and body mass index were collected in Table S1.

### Animal models

For the in vivo experiments, 3-month-old male C57BL/6 mice (*n* = 20) were used. All experiments were approved by the Animal Care and Use Committee of Zhujiang Hospital, Southern Medical University (LAEC-2020-121). All animal experimental operations were in accordance with the approved study protocol and relevant laboratory animal ethics regulations. Destabilization of the medial meniscus (DMM) surgery was performed in mice to induce experimental OA, according to previous studies [27]. Mice were divided into four groups (5 mice per group) by random assignment: Sham+Ad-Ctrl, Sham+Ad-circNFKB1, DMM + Ad-Ctrl, and DMM + Ad-circNFKB1 groups. Similar to a previous study [28, 29]. intra-articularly (IA) injection of adenovirus (1 × 10^9^ plaque-forming units (PFUs) in a total volume of 10 µL) expressing circNFKB1 (Ad-circNFKB1) or its antisense sequence (Ad-Ctrl) was operated every two weeks. To better reflect the biological effect caused by circNFKB1 instead of exogenous circRNA, antisense sequence of circNFKB1 was used as control, which could specifically show the biological role of circNKFB1. Mice were sacrificed 8 weeks after DMM surgery or first IA injection. Knee articular cartilage was further subjected to histological and biochemical analysis.

### Cell isolation and culture

Primary chondrocytes were isolated from human cartilages, similar to described previously [30]. Briefly, cartilage tissues were washed with sterile PBS containing 1% dual antibody three times and cut into small fragments (0.5 mm^3^). The clipped cartilage fragments were then digested with 0.25% trypsin (containing 0.02% EDTA) for 30 min, followed by 0.2% type II collagenase for 16–24 h in a 37 °C thermostatic oscillator. Filtration of the digest with a 70 µm pore size mesh to obtain primary chondrocytes. The isolated primary chondrocytes were then cultured in F12/Dulbecco’s Modified Eagle’s Medium (DMEM; Gibco) with 10% fetal bovine serum (FBS; Invitrogen) and 1% penicillin–streptomycin (Sigma) at 37 °C in 5% CO_2_. The chondrocytes were digested by 0.25% trypsin and 1 mM EDTA and passed for expansion at the growth confluence. Primary chondrocytes at 80% confluence were used for the experiments. In some experiments, 10 ng/mL of IL-1β (Sigma) or PBS was used to treat the cells.

### RNA-sequencing analysis

Chondrocytes stimulated with inflammatory cytokines (10 ng/mL IL-1β) for 4 h were collected for total RNA extraction with TRIzol reagent (Takara Bio). Samples were analyzed by Novel Bioinformatics Co. Ltd with next-generation sequencing. For each human chondrocyte sample, ~2 × 10^5^ cells were used for transcriptome analysis. The next-generation sequencing data used in the study (GSE158875) are available in a public repository from NCBI.

### In situ hybridization (ISH)

The expression and cellular location of circNFKB1 in cultured human chondrocytes was detected by RNA fluorescent in situ hybridization (FISH) as previously described [31]. The RNA-FISH experimental procedures were based on the detailed protocol from a Ribo^TM^ Fluorescent In Situ Hybridization Kit (RiboBio). To detect circNFKB1, RNA-FISH probes against circNFKB1 and all relevant reagents were purchased from RiboBio company. U6 probes and 18 S probes were used as a nucleus and cytoplasm control. In brief, cells were cleansed in PBS and then fixed in fixatives(3.7% formaldehyde solution with RNase-free PBS) for 10 min at room temperature. Subsequently, cells were permeabilized with 0.1% Triton X-100 for 5 min at 4 °C. Cy3-labeled probes were used to perform hybridization at 37 °C under light-proof conditions. Following overnight hybridization, the cells were washed for 15 min in 4× SSC at 42 °C, 2× SSC at 42 °C, and then at 42 °C, 30 min in 2× SSC. After washing, the cell nucleus was stained with 4′,6-Diamidino-2-phenylindole dihydrochloride (DAPI). For colocalization studies, after RNA-FISH, antibodies were added to detect RNA and protein colocalization. Stained samples were mounted with Fluoromount-G for confocal imaging. Laser scanning confocal microscopy (Carl Zeiss, Jena, Germany) was used to visualize the samples described above.

For the measurement of circNFKB1 in human cartilage samples and mouse knee joints, in situ hybridization was operated based on the detailed protocol from a In Situ Hybridization Kit (Boster company). Briefly, sections of the paraffin-embedded cartilage tissue or knee joint were digested with trypsin (ZSGB-BIO) and fixed in 4% paraformaldehyde, then hybridized with circNFKB1 probe at 42 °C overnight. Samples were then washed at 37 °C for 10 min in 2× SSC, 15 min in 0.5× SSC, and 15 min in 0.2× SSC, and were dyed with blue tetrazolium/5-bromo-4-chloro-3-indolylphosphate (Boster) at last. Staining scores were calculated according to the proportion of the circNFKB1-positive cells under a 20× objective.

### RNA isolation, reverse transcription, and quantitative real-time polymerase chain reaction (qRT-PCR)

Total cellular RNA was isolated from cultured chondrocytes using TRIzol (Takara Bio). PrimeScript RT reagent kit (Takara Bio) was used to reverse RNA transcripts into complementary DNA (cDNA). qRT-PCR was performed to measure the RNA expression levels using SYBR Premix Ex Taq (Takara Bio) according to the manufacturer’s instructions. Data were collected and analyzed on a 7500 Real-Time PCR detection system (Applied Biosystems). GAPDH was used as the reference gene. The primer sequences for each gene are listed in Table S2.

### Cell cytoplasm and nucleus fractionation

To isolate cytoplasmic and nuclear RNAs from human primary chondrocytes, PARIS kit (Thermo Fisher Scientific, AM1921) was used according to the manufacturer’s instructions, as described previously [32]. Cytoplasmic and nuclear RNA transcripts were first reversed into cDNA and evaluated by qRT-PCR. The percentages of nuclear and cytoplasmic RNA were assessed according to the qRT-PCR data.

### RNase R/Actinomycin d

For RNase R experiment, total RNA (at least 2 μg) extraction from human chondrocytes was used to incubate with 3 U/μg RNase R (Epicentre Technologies, Madison, WI, USA) for 15 min at 37 °C. All the experiments were carried out without RNA enzyme, and the reaction solution was added on ice. For actinomycin D experiment, about 2 × 10^5^ human chondrocytes were treated with 2 ng/ml actinomycin D and the cells were collected at the indicated time points. CircNFKB1 and NFKB1 mRNA expression was measured by qRT-PCR.

### RNA interference and transient infection

Interference of circRNA were conducted using the antisense oligonucleotides (ASOs) designed and synthesized in GenePharma (China). Two independent ASOs with different sequences target the back-splice junction of circNFKB1. SiRNA for human ENO1 were designed and synthesized by GenePharma (China). Human chondrocytes were seeded in 12-well plates at a density of 1.5 × 10^5^ per well. Chondrocytes were transfected with ASOs/siRNA or its NC (40 nM) using Lipofectamine 3000 reagent following the manufacturer’s instructions. After 48 h, chondrocytes were treated with IL-1β (10 ng/mL) for another 24 h, and then cells were harvested for further analysis. The sequence of ASOs and siRNA for indicated RNAs are listed in Table S3.

### Adenovirus infection

The circNFKB1 overexpression adenovirus [1 × 10^12^ PFUs/ml] and its antisense control were constructed and packaged by Geneseed Biotech (Guangzhou, China). Adenovirus infection in primary chondrocytes was performed as described previously [29]. Briefly, P2 chondrocytes in good condition were selected and seeded into 12-well plate with a cell density of 1 × 10^5^ cells/well. Adenovirus infection could be carried out when the degree of cell fusion was 50–60%. 200 MOI dose of adenovirus and virus transduction reagent were added to the cells, and the well plates were cultured in a 5% CO_2_ incubator at 37 °C for 24 h. After the incubation, the medium was replaced with DMEM/F12 medium containing 10% FBS. The percentage of GFP-positive cells was calculated with an inverted fluorescence microscope (Nikon, Tokyo, Japan) to evaluate the adenovirus transduction efficiency. Adenovirus-infected cells were harvested after the relevant treatment at indicated time points for further analysis.

### Western blotting and antibodies

Cultured cells were harvested, washed with cold PBS, and lysed in RIPA buffer (Beyotime) containing 1% PMSF (Sigma). Primary antibodies against MMP3 (Abcam, ab52915), MMP13 (Proteintech, 18165-1-AP), p-p65 (Cell Signaling, 3033), total p65 (Cell Signaling, 8242), NFKB1 (Proteintech, 14220-1-AP), ENO1 (Proteintech, 11204-1-AP), GAPDH (CWBIO, CW0100M) were used. Normally, primary antibodies were diluted 1:1000 and incubated with the membranes at 4 °C overnight. After washes with TBST at least three times, secondary antibodies such as horseradish peroxidase (HRP)-conjugated anti-rabbit IgG (Boster, BA1054) or anti-mouse IgG (Boster, BA1050) were diluted at 1:1,000 and incubated with the membranes at room temperature for 1 h. Immobilon Western chemiluminescent HRP substrate (Millipore) was used to visualize the membranes. To quantify band intensities, ImageJ software (National Institutes of Health, Bethesda, MD, USA) was used for the analyses. GAPDH was used to normalize the intensity of each band.

### Immunofluorescence staining

Normally, P2 human primary chondrocytes were selected for this experiment. Briefly, the sterile cell plate is carefully put into the 24-well plate, and human chondrocytes were seeded on sterile glass coverslips at the cell density of 3 × 10^4^ per well. When cells were treated with relevant stimulation, growth medium was aspirated and 4% paraformaldehyde was added to fix the cells for 20 min at room temperature. After washes with PBS, 0.2% Triton X-100 was added for 5 min at room temperature to permeabilize the cells, followed by 5% BSA for blocking for 30 min. Primary antibody (1:200) against ENO1 (Proteintech, 11204-1-AP), Aggrecan (Proteintech, 13880-1-AP) and Collagen II (Abcam, ab34712) was added to incubate with the cells overnight at 4 °C. After cells were washed, fluorescent Alexa Fluor^®^ 555-conjugated anti-rabbit IgG (1:500; Cell Signaling, 4413) was used to incubate for 1 h in dark at room temperature. The cell nuclei was stained by 4′, 6-diamidino-2-phenylindole (DAPI). Images were obtained using Nikon Ti2-E at wavelengths of 555 nm (red) and 405 nm (blue, DAPI).

### RNA pulldown and mass spectrometry assays

As described previously [33], biotin-labeled circNFKB1 probes targeting its back-splice and its antisense probes were purchased from GenePharma for RNA pulldown assays. The probe sequences are shown in Table S3. RNA pulldown was performed according to the detailed protocol form the Pierce Magnetic RNA-Protein Pulldown Kit (Thermo Fisher Scientific). Human chondrocytes were cross-linked with 3% formaldehyde at 37 °C for 30 min, and then extracted with co-IP lysate on the ice for 1 h. Extracts were mixed with biotinylated probes and hybridized overnight in a 37 °C hybridization incubator for the probes to fully bind to the proteins. After hybridization, streptavidin magnetic beads were added and incubated for 1 h in a 37 °C hybridization incubator, and then washed with co-IP lysate 5 times to adequately wash away non-specific binding proteins. Subsequently, biotin elution buffer was added and incubated at 37 °C for 1 h to elute the bead-bound proteins. The pulldown proteins were separated using SDS-PAGE followed by silver staining using Pierce Silver Stain for Mass Spectrometry (Thermo Fisher Scientific). Differential bands specifically enriched by circNFKB1 were collected for mass spectrometry. Data are available via ProteomeXchange with identifier PXD033469. Finally, the pulldown proteins were also detected by Western blotting.

### Micro-CT analysis

All knee joint specimens were fixed in 4% paraformaldehyde and scanned using a high-resolution Micro-CT Scanner (ZKKS-MCT-Sharp scanner, Caskaisheng, China). The scanner was set to a voltage of 70 kV, current of 100 μA, exposure time of 100 ms, and resolution of 10 μm. Three-dimensional reconstruction and images visualization of the knee joint were operated with Micro-CT Analysis Software (Micro-CT Reconstruction, ZKKS-Micro-CT 4.1). The region of interest on the sample images was defined to cover all osteophytes.

### Histological analysis, scoring system, and immunohistochemistry

Human cartilage tissues were fixed in 4% paraformaldehyde, embedded in paraffin, and made in sections. Mouse knee joints were fixed in 4% paraformaldehyde, decalcified in 0.5 µM EDTA, and embedded in paraffin. For Safranin-O and fast green staining, all sections were deparaffinized in xylene, hydrated with graded ethanol, and stained with Safranin-O and fast green (Sigma). Cartilage destruction, synovitis, and osteophyte formation were scored by three observe under blinded conditions using the OARSI (grade 0–6), synovitis (grade 0–3), and osteophyte (grade 0–3) scoring system. The OARSI, synovitis, and osteophyte scores are presented as the mean of the maximum score in each mouse, and each representative Safranin-O-stained image was selected from the most advanced lesion among serial sections. For immunohistochemistry, the slides were treated with EDTA repair solution in a boiling water bath for 30 min, and blocked in 5% BSA (MCE) for 1 h. Primary antibodies against MMP3 (1:100; Abcam, ab52915), MMP13 (1:100; Proteintech, 18165-1-AP), NFKB1 (Proteintech, 14220-1-AP), and p-p65 (Cell Signaling, 3033) were added into the slides at 4 °C overnight. The next day, the slides were washed with PBS thrice and incubated with secondary fluorescence antibodies for 1 h at room temperature. DAPI was used to stain the nuclei. Nikon Ti2-E was used to acquire the fluorescence images.

### Statistical analysis

GraphPad Prism 7 (GraphPad, La Jolla, CA, USA) was used to conduct the statistical analyses, and error bars indicate SD or 95% confidence intervals. For qRT-PCR, western blotting, and immunostaining, mean ± standard deviation (SD) was used to present the data; For OARSI score, synovitis score, and osteophyte score, mean ± 95% confidence interval (CI) was used to express them. All experiments were repeated at least three times. In all figure legends, the information about the statistical details is indicated clearly. *P* < 0.05 were considered statistically significant.

## Results

### circNFKB1 is upregulated in inflamed chondrocytes and osteoarthritic cartilage

**Fig. 1:**
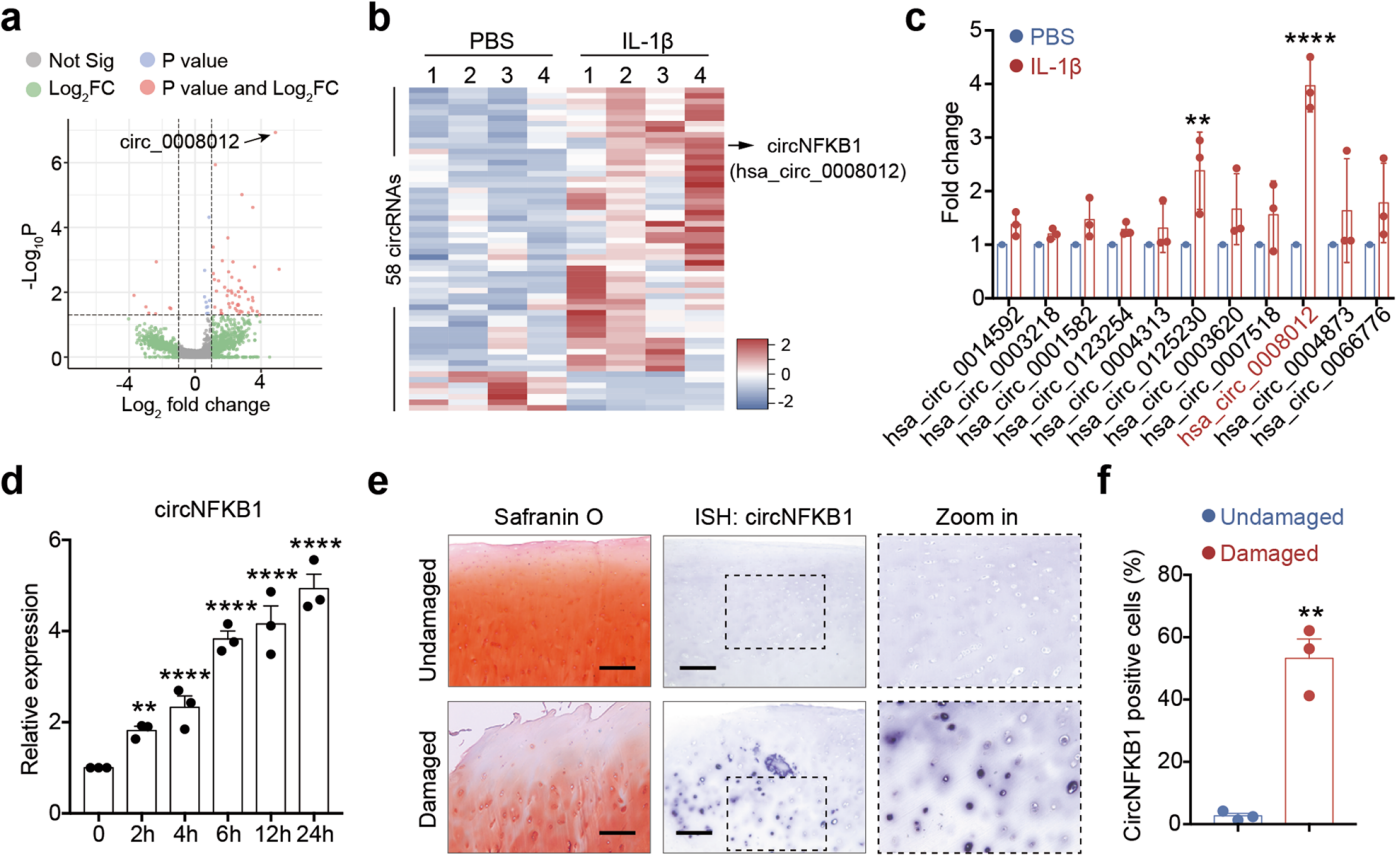
circNFKB1 is upregulated in inflamed chondrocytes and osteoarthritic cartilage. **a** Volcano plot shows the circRNAs expression profiling in human chondrocytes (HCs) stimulated with IL-1β. **b** Heat map of deregulated circRNAs in PBS or IL-1β treated HCs. **c** qRT-PCR validates potential increased circRNAs from sequencing data in IL-1β-stimulated HCs. **d** Expression of circNFKB1 assayed by qRT-PCR within HCs cultured with IL-1β for various time points. **e** Representative images show safranin-O/fast green staining (**e**, left panels) of sulfated proteoglycans and in situ hybridization (ISH) staining (**e**, right panels) of circNFKB1 in undamaged and damaged regions of human osteoarthritic cartilage. **f** the quantification of circNFKB1-positive cells in ISH staining sections. **c**–**e**, *n* = 3 biologically independent experiments, mean values ± SD are shown, and *p* values were calculated by two-tailed Student’s *t* test (**c**, **f**) or one-way ANOVA followed by Sidak’s multiple comparison test (**d**). **p < 0.01, ****p < 0.0001. **e** representative images are shown.

Accumulating evidences demonstrate that NF-κB signaling pathway plays an essential role in the pathogenesis of OA [34]. To identify circRNAs that are potentially involved in this inflammatory signaling, we first generated a circRNA profiling database by circRNA deep-sequencing of ribosomal RNA-depleted total RNA from four inflammatory cytokines (IL-1β)-stimulated human chondrocytes (HCs) and four untreated HCs. Comparative analysis revealed that 58 circRNAs were dysregulated by over twofold in inflamed chondrocytes (Fig. 1a). Of note, 53 circRNAs could be identified and annotated in circRNA database (circBase, http://www.circbase.org/) while five unannotated candidates were firstly discovered in our circRNA profiling (Table S4). Further analysis revealed that 51 circRNAs were significantly upregulated while another seven circRNAs were downregulated markedly (Fig. 1b). According to our qRT-PCR validation, has_circ_0008012, derived from exon 2-3-4-5 of the NFKB1 transcript, was the most significantly upregulated circRNA, which was termed circNFKB1 hereafter (Fig. 1c). We further confirmed that the expression of circNFKB1 under the stimulation of IL-1β (10 ng/mL) was upregulated gradually with the increase of stimulation time (Fig. 1d), suggesting that circNFKB1 may exhibit a certain biological function in pathological process of chondrocyte degradation. To explore the relationship between circNFKB1 and OA, RNA-ISH was performed to detect the expression of circNFKB1 in damaged and undamaged cartilages. The result showed that circNFKB1 was increased in OA damaged cartilage (Fig. 1e, f). In conclusion, we found an OA-related circRNA, circNFKB1, which was increased in inflamed chondrocytes and osteoarthritic cartilage.

### Characterization of circNFKB1 in human chondrocytes

**Fig. 2:**
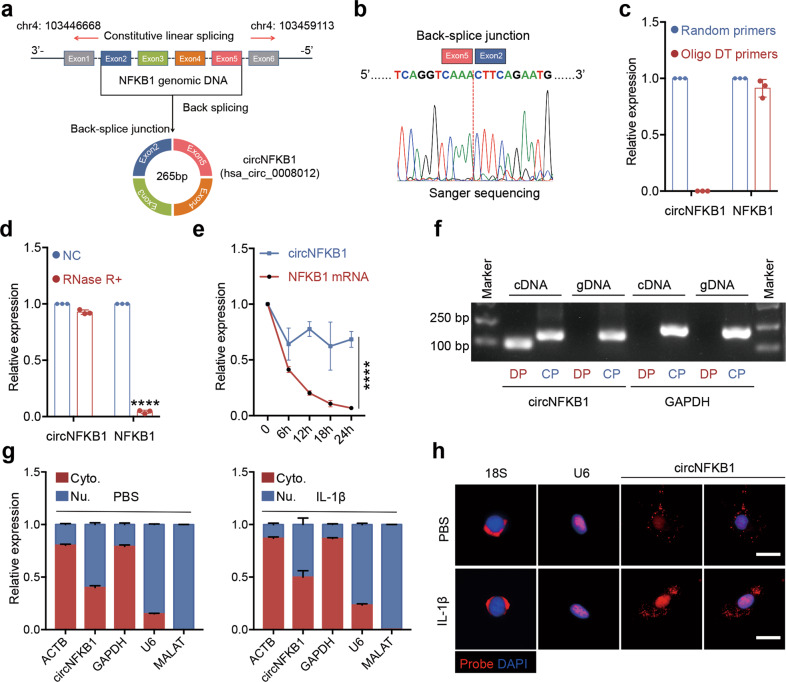
Characterization of circNFKB1 in human chondrocytes. **a** Schematic illustration of circNFKB1 formation via the circularization of exons 2-3-4-5 in NFKB1 gene. **b** The back-splice junction sequence of circNFKB1 was validated by Sanger sequencing. **c** qRT-PCR results of circNFKB1 and linear NFKB1 mRNA using random primers or oligo DT primers. **d** qRT-PCR results of circNFKB1 and linear NFKB1 treated with or without RNase R. **e** Relative abundance of circNFKB1 and linear NFKB1 in HCs treated with actinomycin D. RNAs were measured via qRT-PCR at the indicated time. **f** PCR products of divergent or convergent primers and HCs cDNA or gDNA were validated by gel electrophoresis. **g** Subcellular distribution of circNFKB1 was detected by nuclear mass separation assay in HCs. MALAT1 and U6 acted as nuclear controls whereas GAPDH and ACTB acted as cytoplasm controls. **h** RNA-FISH images showing intracellular localization of circNFKB1 (red) in HCs. U6 and 18 S acted as nuclear and cytoplasm controls, respectively. Scale bar, 10 μm. **c**–**e**, **g** *n* = 3 biologically independent experiments, mean values ± SD are shown, and *p* values were calculated by two-tailed Student’s *t* test (**d**, **e**). ****p < 0.0001. **b**, **f**, **h**, representative images are shown.

Compared with other noncoding RNAs, circRNAs have its unique molecular characteristics, which are the basis of their biological function and mechanism of bioactivity [35]. According to the database of circBase, circNFKB1 is a 265 bp circRNA formed by back splicing of exons 2-3-4-5 of NFKB1 gene on chromosome 4 (Fig. 2a). To clarify the sequence of the back-splice junction of circNFKB1, we conducted sanger sequencing and the result showed that exon 2 and 5 of NFKB1 were connected together to form the junction of circNFKB1 (Fig. 2b). CircNFKB1 could not be detected by oligo DT primers (Fig. 2c). This result indicated that circNFKB1 had no poly-A tail. Linear RNA could be digested by RNA exonuclease and degraded naturally, whereas circRNA could tolerate digestion and degradation due to its special circular structure. To demonstrate circNFKB1 stability, RNase R and actinomycin D were used to digest RNA transcripts and inhibit RNA synthesis in HCs, respectively. The results showed that circNFKB1 was more stable than its linear form NFKB1 mRNA (Fig. 2d, e). Due to the linear nature, linear mRNA could be amplified by convergent primers in both genomic DNA (gDNA) and cDNA, but not be amplified by divergent primer in gDNA or cDNA. Different from linear mRNAs, circRNAs could be amplified by divergent primers in cDNA due to their unique ring structure formed by back splicing of pre-mRNA [36]. Reverse transcription PCR (RT-PCR) analyses showed that both *GAPDH* mRNA and circNFKB1 could be amplified by convergent primers in cDNA and gDNA, but only circNFKB1 could be amplified by divergent primers from cDNA (Fig. 2f). This result indicated that circNFKB1 was circRNA rather than circular DNA generated from the genome. In addition, we examined the relative abundance of circNFKB1 in the nucleus and cytoplasm via qRT-PCR of the nuclear and cytoplasmic fractionations. The results indicated that circNFKB1 was expressed in both chondrocyte cytoplasm and nucleus equivalently (Fig. 2g). We also confirmed the localization of circNFKB1 by RNA-FISH and obtained similar results (Fig. 2h). Taken together, our study show circNFKB1 is a circular RNA, derived from *NFKB1* gene, and expressed in the cytoplasm and nucleus of chondrocytes.

### Knockdown of circNFKB1 suppresses IL-1β-induced ECM catabolism in human chondrocytes

**Fig. 3:**
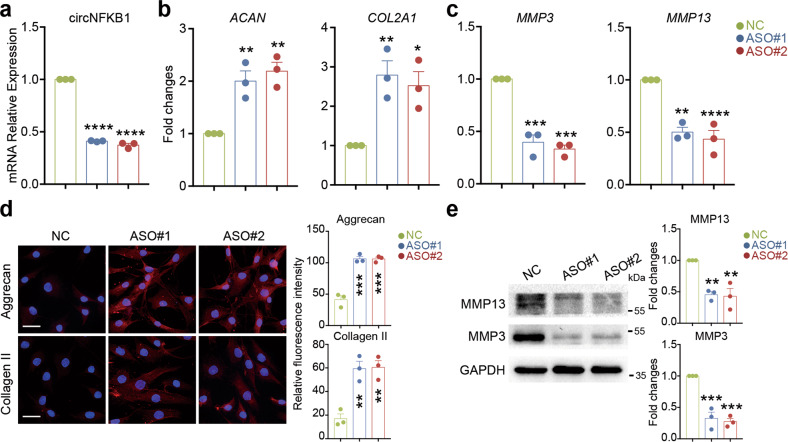
Knockdown of circNFKB1 suppresses IL-1β-induced ECM catabolism in human chondrocytes. **a** qRT-PCR analysis showing circNFKB1 RNA levels in circNFKB1 inhibited HCs. **b**, **c** qRT-PCR analysis mRNA levels of *ACAN*, *COL2A1*, *MMP3,* and *MMP13* in circNFKB1 inhibited HCs with IL-1β stimulation. **d** Representative immunofluorescence images of of Aggrecan and Collagen II protein levels in chondrocytes after circNFKB1 knockdown. Scale bars, 25 μm. **e** Western blotting analysis of MMP3 and MMP13 expression in circNFKB1 knockdown group. **a**–**e** *n* = 3 biologically independent experiments, mean values ± SD are shown, and *p* values were calculated by one-way ANOVA followed by Sidak’s multiple comparison test. *p < 0.05, **p < 0.01, ***p < 0.001, ****p < 0.0001. **d**, **e**, representative images are shown.

To elucidate the biological role of circNFKB1 in chondrocyte degradation, we performed RNA interference experiments in HCs using two independent RNase H-based ASOs targeting the back-splice junction of circNFKB1. qRT-PCR demonstrated that two specific ASOs could knockdown circNFKB1 effectively (Fig. 3a). Furthermore, knockdown of circNFKB1 significantly increased the expression of ECM anabolism genes *ACAN* and *COL2A1* whereas circNFKB1 knockdown decreased the expression of matrix-degrading enzyme genes *MMP3* and *MMP13* in IL-1β-treated HCs (Fig. 3b, c). Immunofluorescence staining confirmed that ASO-mediated circNFKB1 knockdown significantly increased the expression levels of matrix proteins Aggrecan and Collagen II (Fig. 3d). Meanwhile, western blotting demonstrated that MMP3 and MMP13 protein levels were decreased in circNFKB1 knockdown group (Fig. 3e). Collectively, our data reveal that knockdown of circNFKB1 suppresses IL-1β-induced ECM catabolism in HCs.

### Enhanced expression of circNFKB1 initiates ECM catabolism and promotes IL-1β-induced chondrocytes degradation

**Fig. 4:**
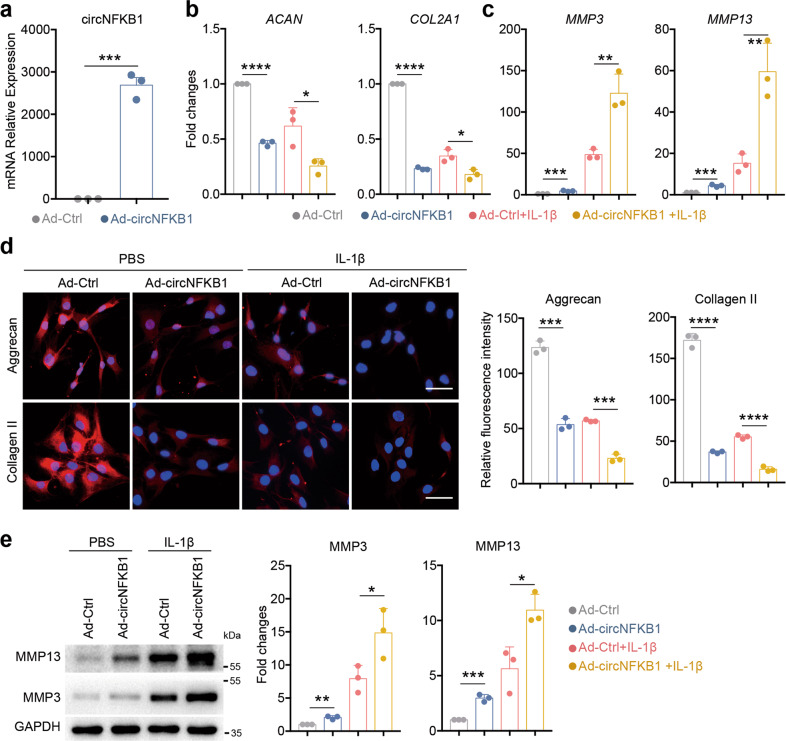
Overexpression of circNFKB1 initiates ECM catabolism and promotes IL-1β-induced chondrocytes degradation. **a** qRT-PCR analysis showing circNFKB1 RNA levels in circNFKB1 overexpressed HCs. **b**, **c** qRT-PCR analysis mRNA levels of *ACAN*, *COL2A1*, *MMP3,* and *MMP13* in circNFKB1 overexpressed HCs. **d** Representative immunofluorescence images and quantitative data of Aggrecan and Collagen II protein levels in chondrocytes after circNFKB1 overexpression. Scale bars, 25 μm. **e** Western blotting analysis of MMP3 and MMP13 expression in circNFKB1 overexpressed chondrocytes. **a**–**e**, *n* = 3 biologically independent experiments, mean values ± SD are shown, and *p* values were calculated by two-tailed Student’s *t* test. *p < 0.05, **p < 0.01, ***p < 0.001, ****p < 0.0001. **d**, **e** representative images are shown.

Next, we conducted circNFKB1 enhanced expression through overexpressed adenovirus in HCs to further explore its roles. qRT-PCR demonstrated that overexpressed adenovirus could enhance circNFKB1 expression significantly (Fig. 4a). Moreover, overexpressed circNFKB1 significantly decreased ECM anabolic genes of *ACAN* and *COL2A1* expression whereas circNFKB1 overexpression increased matrix-degrading enzyme genes of *MMP3* and *MMP13* expression in HCs (Fig. 4b, c). Immunofluorescence staining further confirmed that circNFKB1 overexpression significantly decreased the expression levels of matrix proteins Aggrecan and Collagen II (Fig. 4d). Meanwhile, western blotting demonstrated that MMP3 and MMP13 protein levels were dramatically increased in circNFKB1 overexpressed group (Fig. 4e). Taken together, these data suggest that overexpression of circNFKB1 initiates ECM catabolism and promotes IL-1β-induced chondrocytes degradation.

### circNFKB1 aggravates OA progression in DMM surgery-induced OA mouse model

**Fig. 5:**
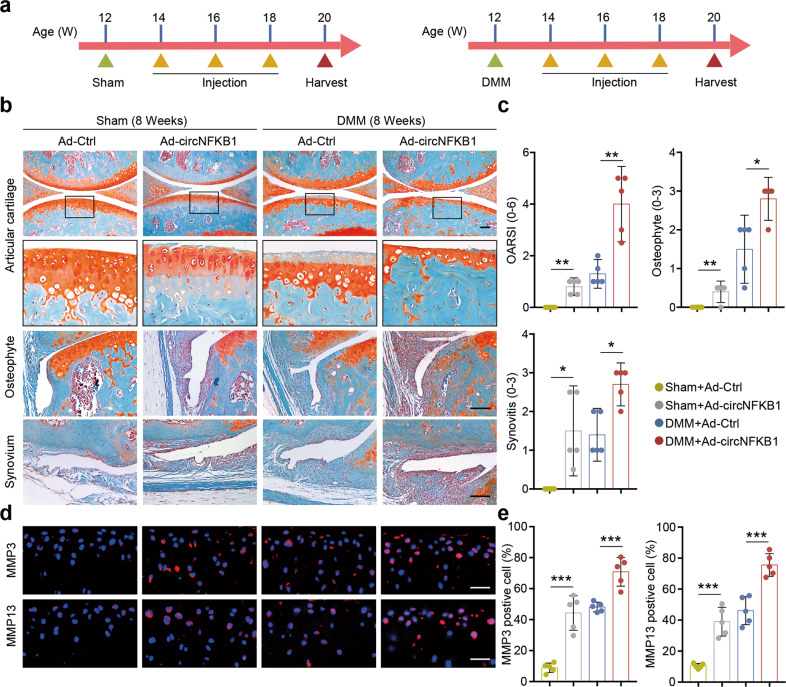
Overexpression of circNFKB1 aggravates OA progression in DMM surgery-induced OA mouse model. **a** Schematic illustration of circNFKB1 delivery schedules in sham mice or DMM mice. **b** Safranin-O/fast green staining of the cartilage (upper panels), osteophyte (middle panels), and synovium (lower panels) (*n* = 5 mice). Scale bars, 100 μm. **c** Scoring of OARSI, Osteophyte, and Synovitis in sham- or DMM-operated mice subjected to IA injection with Ad-Ctrl or Ad-circNFKB1. (*n* = 5 mice per group). Scale bars, 50 μm. **d** Immunofluorescence staining of MMP3 (upper panels) and MM13 (lower panels) of chondrocytes in the indicated groups at 8 weeks after first injection or DMM surgery (*n* = 5 mice). Scale bars, 50 μm. **e** Quantification of MMP3 and MMP13 positive cells were performed according to staining results (*n* = 5 per group). Mean values ± 95% confidence interval (CI) with Mann–Whitney *U* test for **c**. For **e**, mean values ± SD are shown, and *p* values were calculated by two-tailed Student’s *t* test. *p < 0.05, **p < 0.01, ***p < 0.001, ****p < 0.0001, ns, not significant. **b**, **d**, representative images are shown.

To further determine whether circNFKB1 plays a role in cartilage degradation and OA progression in vivo, adenoviral human circNFKB1 (Ad-circNFKB1) and its antisense sequence (Ad-Ctrl) were intra-articularly (IA) administered to sham or DMM-induced OA mice once in 2 weeks (Fig. 5a). RNA-ISH of circNFKB1 was conducted to confirm the in vivo overexpression efficiency. The results showed that injection of Ad-circNFKB1 successfully induced ectopic expression of circNFKB1 in multiple joint tissues, including cartilage, synovium, and meniscus (Fig. S1a). Delivery of Ad-circNFKB1 into mouse knee joint tissues caused significant proteoglycan loss and OA progression, as manifested by cartilage destruction, and synovitis and osteophyte formation; whereas Ad-Ctrl delivery had no detrimental effect on joint (Fig. 5b, c; Fig. S1b). The IA injection of Ad-circNFKB1 aggravated the degradative changes in the cartilage matrix by promoting catabolic response as indicated by immunohistochemistry (Fig. 5d, e). Altogether, these results demonstrate that ectopic expression of circNFKB1 aggravates OA progression in DMM surgery-induced OA mouse model.

### circNFKB1 associates with ENO1 in human chondrocyte nuclei and silencing ENO1 inhibits IL-1β-induced ECM degradation

**Fig. 6:**
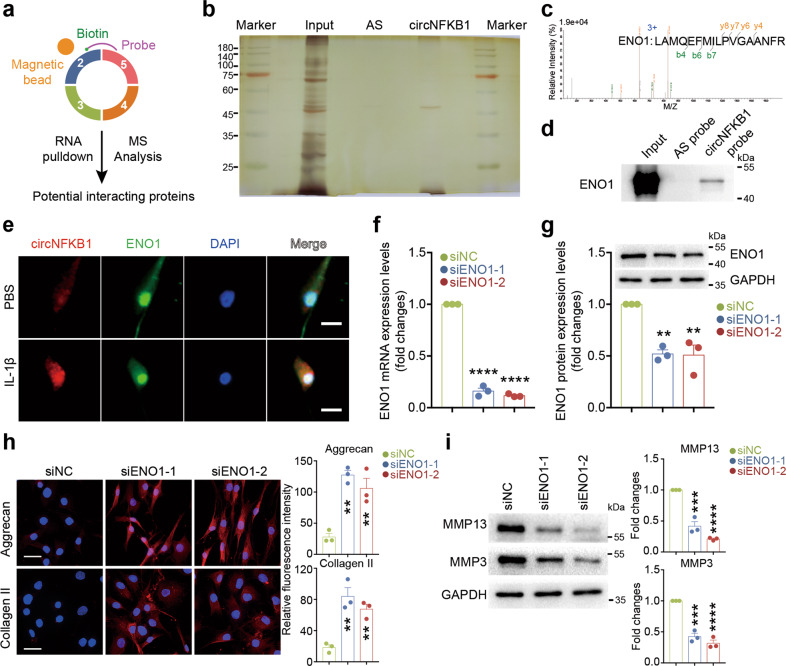
circNFKB1 associates with ENO1 in human chondrocyte nuclei and silencing ENO1 inhibits IL-1β-induced ECM degradation. **a** Schematic illustration of circNFKB1-associated proteins detected by RNA pulldown and MS analysis in human chondrocytes. **b** Silver staining of RNA pulldown assay with circNFKB1 and control probes. AS antisense. **c** Mass spectrometry analysis of circNFKB1-binding proteins after RNA pulldown assay. **d** Western blotting of the interaction between ENO1 circNFKB1 after RNA pulldown. **e** Confocal images showing the colocalization of circNFKB1 and ENO1 in chondrocytes treated with PBS or IL-1β. DAPI, 4′,6-diamidino-2-phenylindole. Scale bar, 10 μm. **f** qRT-PCR analysis showing ENO1 mRNA levels after ENO1 knockdown with siRNAs in HCs. **g** Western blotting analysis showing ENO1 protein levels after ENO1 knockdown. **h** Representative immunofluorescence images of Aggrecan and Collagen II protein levels in chondrocytes after ENO1 knockdown. Scale bars, 25 μm. **i** Western blotting analysis of MMP3 and MMP13 expression in ENO1 knockdown chondrocytes. **f**–**i**, mean values ± SD are shown, and *p* values were calculated by two-tailed one-way ANOVA followed by Sidak’s multiple comparison test. **p < 0.01, ***p < 0.001, ****p < 0.0001, ns, not significant. **b**, **d**, **e**, and **h**, representative images are shown.

Next, we explored the underlying mechanism of circNFKB1-induced chondrocyte degradation. CircRNA-interacting proteins have been attracted more and more attention recently [8]. To clarify potential circNFKB1-interacting protein(s), we performed RNA pulldown assays using biotinylated probe targeting circNFKB1 in HCs cell lysates and analyzed associated protein(s) by mass spectrometry analysis (Fig. 6a). RNA pulldown followed by silver staining revealed a significantly different band over 40 kDa (Fig. 6b). According to mass spectrometry analysis, ENO1 was identified as this specific protein (Fig. 6c). We further confirmed that ENO1 bound with circNFKB1 in RNA pulldown experiments (Fig. 6d). Moreover, we performed circNFKB1 FISH in combination with immunofluorescence of ENO1 and found the colocalization of circNFKB1 and ENO1 in the nucleus of chondrocytes (Fig. 6e). These data present that circNFKB1 interacts with ENO1 to assemble an RNA-protein complex in chondrocytes.

Previous studies reported that ENO1, expressed in arthritic immune cells and synoviocytes, is involved in inflammatory response and maintenance of cell state [25, 26]. However, the expression and role of ENO1 in chondrocytes have not been studied currently. Herein, we found that ENO1 was not significantly increased in IL-1β-stimulated HCs according to the results of qRT-PCR, western blotting, and immunofluorescence staining (Fig. S2). To further elucidate whether ENO1 was involved in regulating chondrocyte degradation, we designed two siRNAs (siENO1-1 and siENO1–2) to inhibit ENO1 expression. As a result, qRT-PCR and western blotting demonstrated that they could efficiently knockdown ENO1 (Fig. 6f, g). More importantly, knockdown of ENO1 significantly increased ECM anabolism markers of Aggrecan and Collagen II expression whereas ENO1 knockdown markedly decreased matrix-degrading enzymes of MMP3 and MMP13 expression in IL-1β-treated HCs (Fig. 6h, i). Altogether, our data indicate that ENO1 plays an important role in the function of circNFKB1.

### circNFKB1 regulates NFKB1 expression and sustains NF-κB signaling pathway

**Fig. 7:**
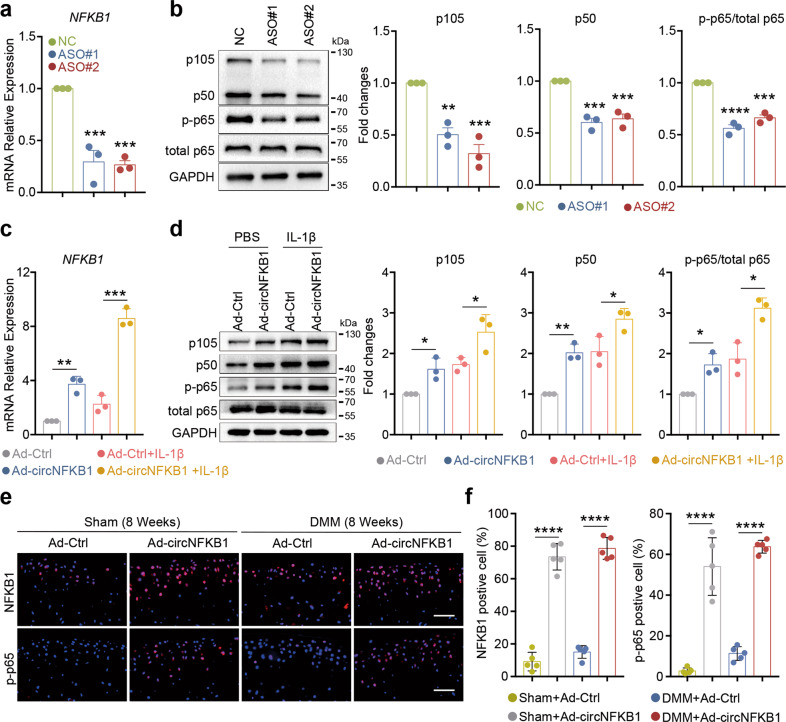
circNFKB1 regulates NFKB1 expression and sustains NF-κB signaling pathway. **a** qRT-PCR analysis showing NFKB1 mRNA levels after circNFKB1 knockdown in HCs. **b** Western blotting analysis showing p105, p50 and p65 phosphorylation protein levels in circNFKB1 knockdown chondrocytes. **c** qRT-PCR analysis showing NFKB1 mRNA levels after circNFKB1 overexpression in HCs. **d** Western blotting analysis showing p105, p50, and p65 phosphorylation protein levels in circNFKB1 overexpressed chondrocytes. **e** Immunofluorescence staining of NFKB1 (upper panels) and p-p65 (lower panels) of chondrocytes in the indicated groups at 8 weeks after first injection or DMM surgery (*n* = 5 mice). Scale bars, 50 μm. **f** Quantification of NFKB1 and p-p65 positive cells were performed according to staining results (*n* = 5 per group). **a**–**d**, **f**, mean values ± SD are shown, and *p* values were calculated by two-tailed Student’s *t* test (**c**, **d**, **f**) or one-way ANOVA followed by Sidak’s multiple comparison test (**a**, **b**). *p < 0.05, **p < 0.01, ***p < 0.001, ****p < 0.0001. **b**, **d**, **e**, representative images are shown.

The gene of *NFKB1* encodes a 105 kD protein, which could be co-translated into a 50 kD protein by the 26 S proteasome. The 105 kD protein (NFKB1/p105) is a Rel protein-specific transcription inhibitor while the 50 kD protein (NFKB1/p50) is a DNA binding subunit of the NF-κB protein complex. Previous studies have demonstrated that circRNAs can regulate the transcription of their parental genes in cis [37, 38]. In this study, we found that circNFKB1 was derived from its parental gene *NFKB1*, so whether circNFKB1 regulated the expression of NFKB1 aroused our interest. To test this hypothesis, we first conducted circNFKB1 knockdown experiments with ASOs and detected the mRNA level of *NFKB1* by qRT-PCR. As a result, circNFKB1 knockdown could significantly decrease the mRNA level of *NFKB1* (Fig. 7a). More importantly, western blotting showed that knockdown of circNFKB1 decreased the protein levels of p105, p50, and IL-1β-induced p65 phosphorylation (Fig. 7b) in HCs. In addition, overexpressing circNFKB1 increased *NFKB1* mRNA expression levels (Fig. 7c). Western blotting showed that overexpression of circNFKB1 upregulated the protein levels of p105, p50, and promoted p65 phosphorylation (Fig. 7d). Moreover, heterotopic overexpression of circNFKB1 in mouse knee articular cartilage increased NFKB1 expression and activated the NF-κB signaling pathway (Fig. 7e, f). Overall, these results suggest that circNFKB1 regulates the expression of its parental gene *NFKB1* and sustains the activation of NF-κB signaling pathway in HCs.

## Discussion

OA has been widely considered a highly heterogeneous disease with multiple phenotypes [39]. Meanwhile, due to the complex OA pathogenesis in that almost all joint tissues are involved, the early diagnosis and therapy of OA have been a challenging issue in clinical practice. Therefore, further exploring the mechanism underlying the cartilage ECM degradation and identifying new molecular targets is of paramount importance to develop diagnostic and therapeutic strategies for this disease. In this study, we found circNFKB1 as a key markedly upregulated circRNA in inflamed chondrocytes and osteoarthritic cartilage. Loss- and gain-of-function in vitro and IA delivery in vivo demonstrated that circNFKB1 promoted the chondrocytes ECM catabolism and aggravated OA progression. We believe that our study uncovers a novel biological role of circNFKB1 in OA progression and represents a promising therapeutic target for the treatment of OA.

NFKB1, the core gene of NF-κB signaling, encodes NFKB1/p105 and NFKB1/p50, which are the members of the Rel-like domain-containing proteins (including RELA/p65, RELB, REL, and NFKB2/p52). These proteins are assembled in pairs to form homo- or heterodimeric complex in the cytoplasm. When the cells are exposed to various intra- and extracellular stimuli such as inflammatory cytokines, mechanical stress, oxidant-free radicals, or even bacterial products, NF-κB will be activated and translocated into the nucleus, initiating RNA transcription by binding at kappa B sites [40]. A recent study reported that downregulated miR-9 in OA cartilage directly binding to NFKB1 mRNA and initiating NFKB1 mRNA decay could facilitate cell proliferation and anti-apoptosis of knee OA chondrocytes [41]. Moreover, a new compound Brazilin isolated from Caesalpinia sappan was reported to effectively protect against catabolic processes in human OA cartilage via the inhibition of NFKB1/p50 [42]. Therefore, targeting NFKB1 could be an effective and promising way to inhibit NF-κB signaling for OA therapy. However, whether there is circRNA generated from NFKB1 and its role in any physiological and pathological processes are not clear at present.

In this study, we identified that circNFKB1 was significantly upregulated in osteoarthritic cartilage, derived from exons 2–5 of *NFKB1* and formed a circular RNA through back-splicing. As with most circRNAs, circNFKB1 shows very high stability and integrity, suggesting that it could be used as stable biomarker for diseases diagnosis and treatment. Through loss-of-function realized by ASOs specifically targeting circNFKB1 junction and gain-of-function realized by adenovirus, we observed that circNFKB1 could significantly regulate the metabolic process of ECM and sustain the activation of NF-κB signaling. Hence, we conclude that the gene of *NFKB1* modulates chondrocytes ECM metabolism not only through its coding protein but also through its originated circRNA. More intriguingly, our study reveals the existence of a new type of circRNA derived from the NF-κB core gene that ultimately regulates the activation of NF-κB signaling pathway. Similarly, a recent study reported that circIKBKB is generated from the *IKBKB* gene, which codes NF-κB kinase subunit beta, facilitating cancer cell metastasis in breast cancer by activating NF-κB pathway [31]. Therefore, our and others’ work suggests further exploration of circRNAs from key genes in the signaling pathway will deepen the understanding of the regulatory network of signaling pathways.

CircRNAs are newly discovered, tissue-specific, and conserved endogenous noncoding RNAs widely existed in mammalian cells. Accumulating evidences have indicated that circRNAs are essential to regulate chondrocytes metabolic homeostasis in the development and progression of musculoskeletal diseases [14]. For example, Shen et al. found that decreased expression of circSERPINE2 in HCs aggravated cell apoptosis and inhibited anabolism of ECM through miR-1271-ERG pathway [15]. Wu et al. reported a novel downregulated circPDE4D in osteoarthritic cartilage acted as a miRNA sponge, thereby regulating miR-103a-3p/FGF18 axis [43]. Shen et al. demonstrated that circCDK14 protected against OA progression by affecting miR-125a-5p/Smad2/TGF-β signaling [17]. This research suggests that dysregulated circRNAs are involved in the pathogenesis of OA mainly by acting as miRNA sponges. In addition, circPDE4B has been demonstrated to participate in post-translational modifications of proteins as a scaffold, thus affecting OA progression [16]. Moreover, oxidative stress-related circRNA circRSU1 and circKIF18A were identified to modulate oxidative stress-triggered inflammation and ECM maintenance. Herein, our study provides a new functional circRNA, circNFKB1, whose mechanism of action is mainly through binding with protein to regulate ECM metabolism. More interestingly, we further unambiguously demonstrated that circNFKB1 regulates the expression of its parental gene *NFKB1* at the transcriptional level. These findings are consistent with previous studies that circRNAs can enhance the transcription of their parental genes *in cis* [38, 44].

The gene of *ENO1* encodes two isoforms, ENO1 and c-myc-binding protein (MBP-1). As a moonlight protein, ENO1 has multiple functions and involves in a variety of cellular processes. The biological role of ENO1 is partly related to its cellular location. Nuclear ENO1 are involved in gene transcription by serving as a DNA binding protein, whereas cytoplasmic ENO1 can regulate mRNA stability, protein translation, and glycolysis cycle [45]. Moreover, ENO1 also could translocate to the cell surface as a membrane protein receptor, which promotes the invasion of monocytes and elicits an enhanced inflammatory response [25, 46]. However, few studies have examined the role of ENO1 in OA pathogenesis. Herein, we found that although ENO1 expression was not significantly changed in inflamed chondrocytes or osteoarthritic cartilage, it was indispensable and essential to maintaining chondrocyte ECM homeostasis. Silence of ENO1 in IL-1β-stimulated chondrocytes suppressed inflammatory cytokine-induced ECM catabolism and rescued impaired ECM anabolism. We also demonstrated that ENO1 bound to circNFKB1 and performed biological functions together. These findings are similar to previous studies that ENO1 was associated with noncoding RNAs (such as tRNA, long noncoding RNAs, and circRNAs) and involved in mitochondrial function, glycolytic reprogramming, and tumor development [22–24, 47]. More interestingly, ENO1 is expressed in both cytoplasm and nucleus of chondrocytes, which is consistent with circNFKB1. Therefore, we speculate that circNFKB1 binding with ENO1 might affect the NK-κB signaling pathway by regulating the transcriptional activity of NFKB1. Further exploration will be conducted to clarify the underlying mechanism in the future.

This study still has limitations. First, it is unavailable for us to determine the function of circNFKB1 in chondrocyte-specific circNFKB1 knockout mice, which would present more conclusive evidence. Second, circNFKB1 interacts with ENO1 in chondrocytes and they promote chondrocytes degradation consistently. Whereas, the detailed mechanism that circNFKB1/ENO1 complex in regulating NF-κB signaling needs further investigation. Future research will be conducted to clarify whether circNFKB1 could be used as a diagnostic marker and therapeutical target for OA in humans.

In summary, our findings identify a new functional ENO1-interacting circNFKB1 that promotes chondrocytes ECM catabolism and exacerbates OA progression by sustaining the activation of NF-κB-signaling pathway. This study provides valuable insights into understanding the regulatory mechanism of NF-κB signaling in chondrocytes and a promising therapeutic target for the treatment of OA.

### Reporting summary

Further information on research design is available in the Nature Research Reporting Summary linked to this article.

## Supplementary information

Supplementary materials

Original full length western blots

Reporting Summary

ARRIVE

## Data Availability

The accession number for the circRNA data reported in this paper is GSE158875 and is publicly accessible at https://www.ncbi.nlm.nih.gov/geo/. The mass spectrometry proteomics data have been deposited to the ProteomeXchange Consortium via the PRIDE partner repository with the dataset identifier PXD033469. All data needed to evaluate the conclusions in the paper are present in the paper and/or the Supplementary Materials.
