# Supplementary material for: Circular RNA circNFKB1 promotes osteoarthritis progression through interacting with ENO1 and sustaining NF-κB signaling
Source: Cell Death Dis. 2022 Aug 9;13(8):695. doi: 10.1038/s41419-022-05148-2 (PMC9363463; doi:10.1038/s41419-022-05148-2)
Supplement: Supplementary file 1 — Supplementary materials [file 41419_2022_5148_MOESM1_ESM.docx]

**Supplementary Materials**

**Table S1. Characteristics of knee OA patients.**

| Characteristics | Values |
| --- | --- |
| Number of patients | 30 |
| Gender (Female), n (%) | 21 (70.0) |
| Weight, kg | 66.8 ± 11.5 |
| Height, cm | 158.0 ± 7.6 |
| Body mass index, kg/m^2^ | 26.7 ± 3.9 |

**Table S2. Primers for qRT-PCR**

| Gene | Forward primer  (5’-3’) | Reverse primer  (5’-3’) |
| --- | --- | --- |
| hsa_circ_0125230 | ACCTGGCTATGAAGGAAGATGG | CAACTCCTCTCTCTTCTGCTTG |
| hsa_circ_0008012 | GCCCATCCCATGGTGGACTA | CTGCCATTCTGAAGTTTGACCT |
| hsa_circ_0123254 | GCAGCACATAAGGATGGCAAA | TTCCAAGCTATCTGTGTTGACCA |
| hsa_circ_0004873 | CAATGCTAGGAGCGATGCAG | AATTTCTTCAACAGGTATCATCCAG |
| hsa_circ_0004313 | AAAATCGGTACCTGTTTCCCA | ATTTCTGGGTGGGACTGCTT |
| hsa_circ_0007518 | CTCTGTACAATGCCGGTGGT | TGAGTCCGCTGAAGAACTCG |
| hsa_circ_0014592 | AAGCACCCAAGGAAAGCACT | ACCAGCATCTTTCTTCGTTTTG |
| hsa_circ_0066776 | AAGTCATCTACATTTCAGGTGCC | AACATTCTTTCCCCATACTGCTG |
| hsa_circ_0001582 | ACGAACATGGGGTTGTTCTCAT | ATCACAGGATGCAGAAGGCAT |
| hsa_circ_0001684 | GCAGAGGAAAATAAGAACACAGT | TCAGCAAGGTTGTATCCACTCT |
| hsa_circ_0003620 | CCAGAGGAACTGACACCATTGA | GCATGCTCAGCTATTACCAGGG |
| GAPDH | GGAGCGAGATCCCTCCAAAAT | GGCTGTTGTCATACTTCTCATGG |
| ACTB | CATGTACGTTGCTATCCAGGC | CTCCTTAATGTCACGCACGAT |
| U6 | CTCGCTTCGGCAGCACA | AACGCTTCACGAATTTGCGT |
| MALAT1 | GTCATAACCAGCCTGGCAGT | CGAAACATTGGCACACAGCA |
| MMP3 | CCTACAAGGAGGCAGGCAAG | CCCGTCACCTCCAATCCAAG |
| MMP13 | TCCTGATGTGGGTGAATACAATG | GCCATCGTGAAGTCTGGTAAAAT |
| ACAN | GTGCCTATCAGGACAAGGTCT | GATGCCTTTCACCACGACTTC |
| COL2A1 | TCCTGCCGTTTCGCTG | CATTATACCTCTGCCCATCCTG |
| NFKB1 | GAAGCACGAATGACAGAGGC | GCTTGGCGGATTAGCTCTTTT |
| ENO1 | TGGTGTCTATCGAAGATCCCTT | CCTTGGCGATCCTCTTTGG |
| circNFKB1-DP | GCCCATCCCATGGTGGACTA | AAACATTTGTTCAGGCCTTCCC |
| circNFKB1-CP | TGGGAAGGCCTGAACAAATG | GTAGTCCACCATGGGATGGG |
| GAPDH-DP | CTGACTTCAACAGCGACACC | CATGGTGTCTGAGCGATGTG |
| GAPDH-CP | GGAGCGAGATCCCTCCAAAAT | GGCTGTTGTCATACTTCTCATGG |

**Table S3. Sequence of oligos**

| Oligo | Sequence 5’-3’ |
| --- | --- |
| NC (ASO) | UCUGAAGUUUGACCUGAGGG |
| ASO#1 | UCUGAAGUUUGACCUGAGGG |
| ASO#2 | CCAUUCUGAAGUUUGACCUG |
| siNC | UUCUCCGAACGUGUCACGUTT |
| siENO1-1 | GCUGGCAACUCUGAAGUCATT |
| siENO1-2 | CCCAGUGGUGUCUAUCGAATT |
| circNFKB1 probe | UCUUCUGCCAUUCUGAAGUUUGACCUGAGGGUAAGA |
| AS probe | UCUUACCCUCAGGUCAAACUUCAGAAUGGCAGAAGA |
| circNFKB1 ISH probe | CATCTTCTGCCATTCTGAAGTTTGACCTGAGGGTAAGACT |
| circNFKB1 FISH probe | TTCTGCCATTCTGAAGTTTGACCTGAGGGTA |

**Table S4. CircRNA expression profile in IL-1β-stimulated human primary chondrocytes.**

| CircBaseID | FoldChange | Style | Normalized_PBS | Normalized_IL-1β | FDR |
| --- | --- | --- | --- | --- | --- |
| *hsa_circ_0014592* | 11.16173079 | up | 0.59216669 | 6.711222482 | 0.014571296 |
| *hsa_circ_0014614* | 0.142713566 | down | 10.4334131 | 1.480416724 | 0.043817499 |
| *hsa_circ_0020028* | 0.345022277 | down | 15.537326 | 5.354173818 | 0.030166588 |
| *Unidentified circRNA1* | 10.56551875 | up | 0.422976207 | 4.564618232 | 0.030279832 |
| *hsa_circ_0007434* | 7.043077592 | up | 0.888250034 | 6.316444689 | 0.007441955 |
| *Unidentified circRNA2* | 11.3666044 | up | 0.59216669 | 6.834590542 | 0.018328446 |
| *hsa_circ_0129006* | 6.170246796 | up | 1.311226241 | 8.142291981 | 0.009633442 |
| *hsa_circ_0002961* | 5.422701894 | up | 1.529763948 | 8.339680878 | 0.03191313 |
| *hsa_circ_0001195* | 3.966651954 | up | 3.954827534 | 15.71709089 | 0.000209723 |
| *hsa_circ_0007785* | 5.897291494 | up | 1.163184569 | 6.908611378 | 0.025074288 |
| *hsa_circ_0001460* | 6.425652583 | up | 2.199476276 | 14.18732694 | 0.013686016 |
| *hsa_circ_0125230* | 34.15620779 | up | 0.246736121 | 8.759132283 | 0.001952743 |
| *hsa_circ_0106834* | 4.310968224 | up | 1.480416724 | 6.415139137 | 0.02883252 |
| *hsa_circ_0001103* | 2.342484217 | up | 44.3702041 | 103.9499276 | 1.16097E-06 |
| *hsa_circ_0109986* | 6.376687509 | up | 1.036291707 | 6.661875258 | 0.045749215 |
| *hsa_circ_0001882* | 2.247595997 | up | 22.78431834 | 51.22241865 | 0.004047888 |
| *hsa_circ_0004996* | 3.138037718 | up | 6.943859395 | 21.81147307 | 0.001064043 |
| *hsa_circ_0007142* | 5.807284668 | up | 1.529763948 | 8.931847568 | 0.037138423 |
| *hsa_circ_0007976* | 4.697130627 | up | 2.566055655 | 12.09006991 | 0.010545925 |
| *hsa_circ_0004412* | 3.843387867 | up | 4.300258103 | 16.5559937 | 0.012691192 |
| *hsa_circ_0135062* | 2.15298295 | up | 28.74828286 | 61.90609267 | 0.002642986 |
| *hsa_circ_0008362* | 0.195491927 | down | 12.91487352 | 2.516708431 | 0.001149551 |
| *hsa_circ_0008012* | 29.29259054 | up | 0.888250034 | 26.30207046 | 1.17031E-07 |
| *hsa_circ_0001582* | 9.353809131 | up | 0.740208362 | 7.007305826 | 0.016572649 |
| *hsa_circ_0003239* | 3.077442273 | up | 5.421145051 | 16.70403537 | 0.045751962 |
| *hsa_circ_0002857* | 0.12644508 | down | 7.87440705 | 0.986944483 | 0.028171016 |
| *hsa_circ_0001508* | 6.170698565 | up | 1.163184569 | 7.229368335 | 0.0250519 |
| *hsa_circ_0002744* | 2.974998153 | up | 6.006262137 | 17.88836875 | 0.036230575 |
| *hsa_circ_0126287* | 6.368134546 | up | 1.529763948 | 9.79542399 | 0.004361454 |
| *hsa_circ_0036429* | 0.361029027 | down | 17.581711 | 6.341118301 | 0.031848247 |
| *hsa_circ_0006935* | 6.877016247 | up | 1.88224412 | 13.00299356 | 0.007211737 |
| *hsa_circ_0113347* | 0.076594917 | down | 6.563180809 | 0.493472241 | 0.012442439 |
| *hsa_circ_0020091* | 12.15200733 | up | 0.63446431 | 7.821535025 | 0.00164915 |
| *hsa_circ_0008365* | 2.449482556 | up | 8.938897171 | 21.91016751 | 0.009197792 |
| *Unidentified circRNA3* | 2.134536457 | up | 37.25010461 | 79.52305169 | 0.000401645 |
| *hsa_circ_0004293* | 4.904790095 | up | 2.150129051 | 10.58497958 | 0.001152836 |
| *hsa_circ_0003218* | 11.3029619 | up | 0.916448448 | 10.46161152 | 2.40611E-05 |
| *hsa_circ_0001684* | 8.28472068 | up | 0.845952414 | 7.081326663 | 0.009561114 |
| *hsa_circ_0119816* | 0.189980812 | down | 7.705216568 | 1.455743112 | 0.045929984 |
| *hsa_circ_0006364* | 3.456642983 | up | 3.412008068 | 11.81866018 | 0.006994051 |
| *hsa_circ_0009964* | 2.684077116 | up | 9.425319809 | 25.31512598 | 0.004424225 |
| *hsa_circ_0005050* | 6.109147418 | up | 0.888250034 | 5.477541878 | 0.023593925 |
| *hsa_circ_0000798* | 4.127765996 | up | 2.072583413 | 8.586416999 | 0.022620166 |
| *hsa_circ_0007786* | 4.092569834 | up | 5.110962499 | 20.94789664 | 0.002339957 |
| *hsa_circ_0066776* | 9.429186213 | up | 0.493472241 | 4.737333516 | 0.042866389 |
| *hsa_circ_0123246* | 4.583861026 | up | 2.220625086 | 10.21487539 | 0.009062879 |
| *hsa_circ_0004313* | 7.189335886 | up | 0.845952414 | 6.143729404 | 0.040334537 |
| *hsa_circ_0067991* | 2.982154548 | up | 5.851170861 | 17.46891734 | 0.044733187 |
| *hsa_circ_0072107* | 4.010763909 | up | 2.114881034 | 8.512396162 | 0.009005499 |
| *hsa_circ_0006674* | 4.684481245 | up | 3.010180672 | 14.13797971 | 0.003433882 |
| *Unidentified circRNA4* | 4.823157547 | up | 1.332375052 | 6.464486361 | 0.021388178 |
| *hsa_circ_0007518* | 11.21445393 | up | 0.444125017 | 5.082764085 | 0.037202259 |
| *hsa_circ_0004873* | 13.60507081 | up | 0.444125017 | 6.168403016 | 0.039131324 |
| *hsa_circ_0087960* | 2.288592263 | up | 61.13416109 | 139.924054 | 0.028681961 |
| *hsa_circ_0006380* | 6.683502724 | up | 0.881200431 | 5.946340508 | 0.038214646 |
| *Unidentified circRNA5* | 5.424519785 | up | 2.220625086 | 12.09006991 | 0.01216813 |
| *hsa_circ_0123254* | 15.06741821 | up | 0.422976207 | 6.513833585 | 0.048959905 |
| *hsa_circ_0003620* | 7.15765604 | up | 2.26997231 | 16.30925757 | 9.6905E-06 |

**Supplementary Figures**


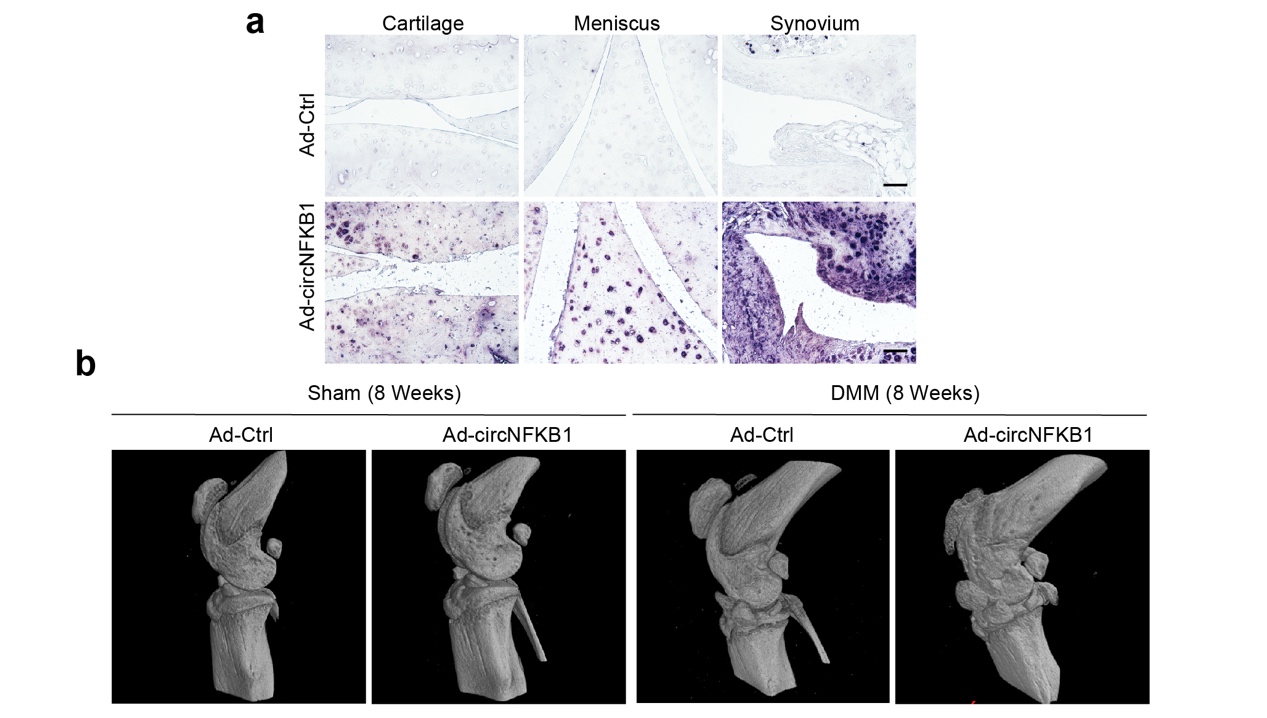


**Fig. S1. Delivery of circNFKB1 aggravates osteoarthritis progression.** (a) In situ hybridization (ISH) of circNFKB1 in sections of cartilage in which Ad-Ctrl or Ad-circNFKB1 was delivered intra-articularly (IA). Scale bar, 100 μm. (b) MicroCT images showing the osteophyte formation in sham- or DMM-operated mice subjected to IA injection with Ad-Ctrl or Ad-circNFKB1. (n = 5 mice per group). For a and b, representative images are shown.


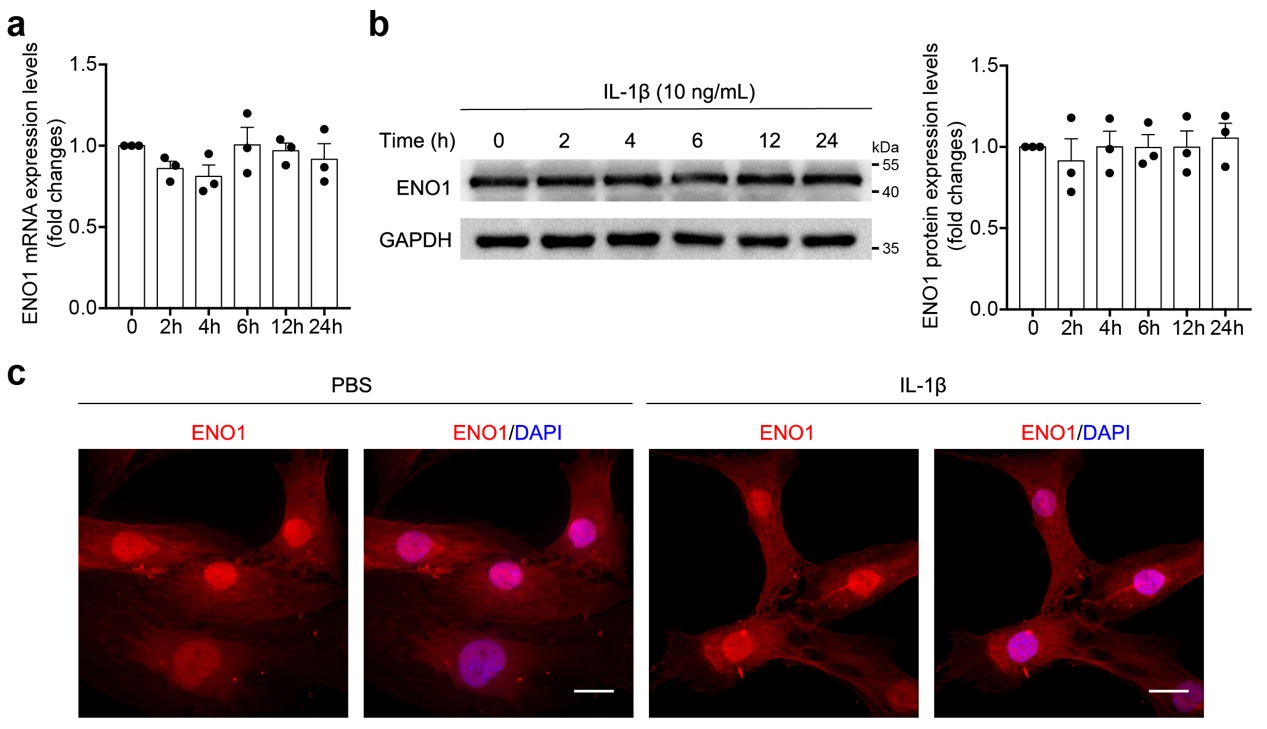


**Fig. S2. ENO1 expression in human chondrocytes.** (a) qRT-PCR analysis showing ENO1 mRNA levels in human chondrocytes with IL-1β stimulation at the indicated time. (b) Western blotting analysis showing ENO1 protein levels n human chondrocytes with IL-1β stimulation at the indicated time. (c) Immunofluorescence staining of ENO1 in human chondrocytes with PBS or IL-1β. For a and b, mean values ± SD are shown, and *p*-values were calculated by two-tailed one-way ANOVA followed by Sidak’s multiple comparison test. For c, representative images are shown.
