# Supplementary figures and images for: Circular RNA circNFKB1 promotes osteoarthritis progression through interacting with ENO1 and sustaining NF-κB signaling
Source: Cell Death Dis. 2022 Aug 9;13(8):695. doi: 10.1038/s41419-022-05148-2 (PMC9363463; doi:10.1038/s41419-022-05148-2)

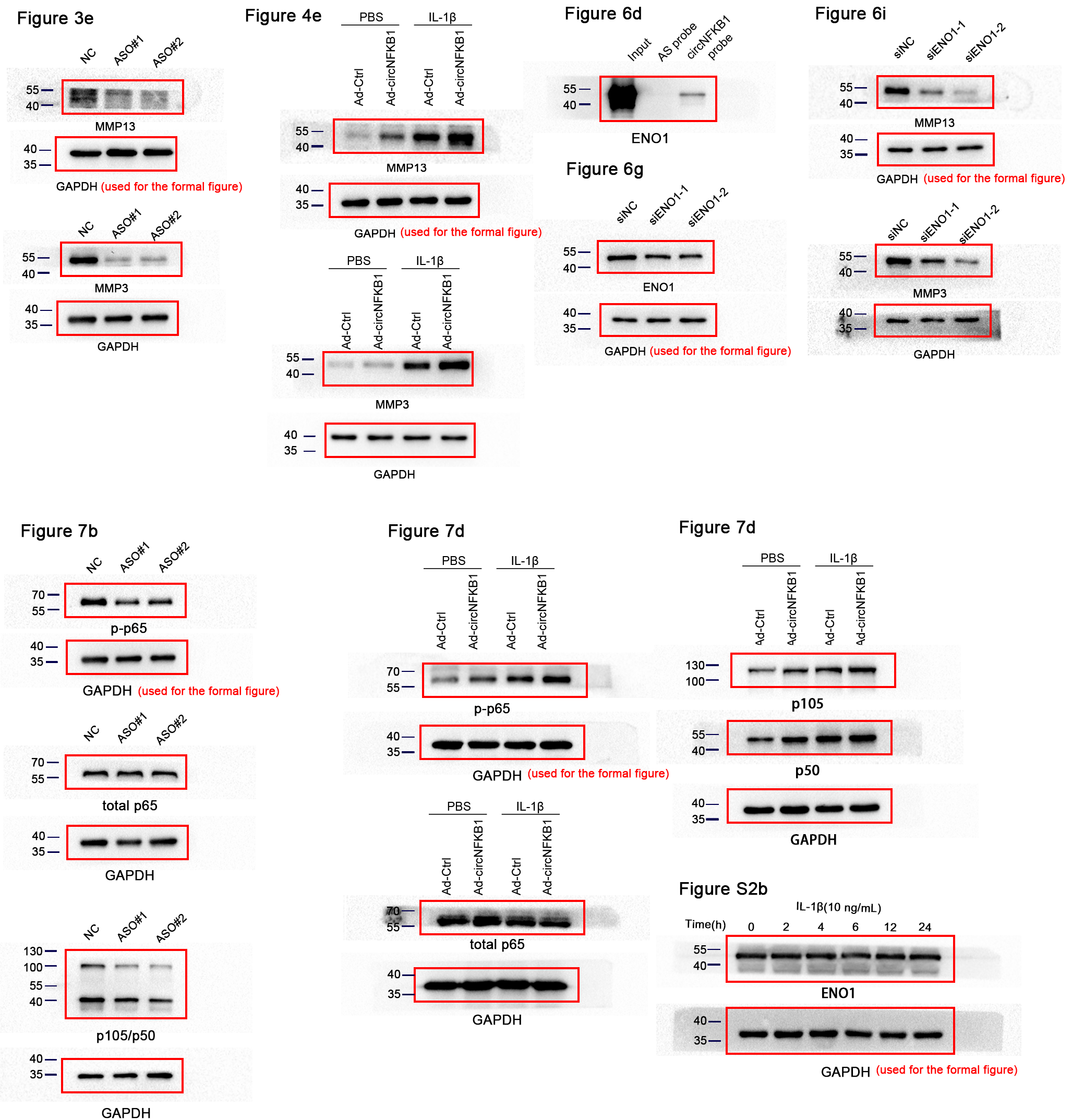

Supplement: Supplementary file 2 — Original full length western blots [file 41419_2022_5148_MOESM2_ESM.tif]
